# Incorporation of PET Metabolic Parameters With Clinical Features Into a Predictive Model for Radiotherapy-Related Esophageal Fistula in Esophageal Squamous Cell Carcinoma

**DOI:** 10.3389/fonc.2022.812707

**Published:** 2022-02-28

**Authors:** Kaixin Li, XiaoLei Ni, Duanyu Lin, Jiancheng Li

**Affiliations:** ^1^ Department of Radiation Oncology, Quanzhou First Hospital Affiliated to Fujian Medical University, Quanzhou, China; ^2^ Department of Radiation Oncology, The First Hospital of Longyan Affiliated to Fujian Medical University, Longyan, China; ^3^ Department of Nuclear Medicine, Fujian Medical University Cancer Hospital, Fujian Cancer Hospital, Fuzhou, China; ^4^ Department of Radiation Oncology, Fujian Medical University Cancer Hospital, Fujian Cancer Hospital, Fuzhou, China

**Keywords:** esophageal squamous cell carcinoma, radiotherapy, esophageal fistula, PET/CT, metabolic parameter

## Abstract

**Purpose:**

To determine whether the addition of metabolic parameters from fluorine-18-fluorodeoxyglucose positron emission tomography/computed tomography (^18^F-FDG PET/CT) scans to clinical factors could improve risk prediction models for radiotherapy-related esophageal fistula (EF) in esophageal squamous cell carcinoma (ESCC).

**Methods and Materials:**

Anonymized data from 185 ESCC patients (20 radiotherapy-related EF-positive cases) were collected, including pre-therapy PET/CT scans and EF status. In total, 29 clinical features and 15 metabolic parameters from PET/CT were included in the analysis, and a least absolute shrinkage and selection operator logistic regression model was used to construct a risk score (RS) system. The predictive capabilities of the models were compared using receiver operating characteristic (ROC) curves.

**Results:**

In univariate analysis, metabolic tumor volume (MTV)_40% was a risk factor for radiotherapy (RT)-related EF, with an odds ratio (OR) of 1.036 [95% confidence interval (CI): 1.009–1.063, *p* = 0.007]. However, it was excluded from the predictive model using multivariate logistic regression. Predictive models were built based on the clinical features in the training cohort. The model included diabetes, tumor length and thickness, adjuvant chemotherapy, eosinophil count, and monocyte-to-lymphocyte ratio. The RS was defined as follows: 0.2832 − (7.1369 × diabetes) + (1.4304 × tumor length) + (2.1409 × tumor thickness) – [8.3967 × adjuvant chemotherapy (ACT)] − (28.7671 × eosinophils) + (8.2213 × MLR). The cutoff of RS was set at −1.415, with an area under the curve (AUC) of 0.977 (95% CI: 0.9536–1), a specificity of 0.929, and a sensitivity of 1. Analysis in the testing cohort showed a lower AUC of 0.795 (95% CI: 0.577–1), a specificity of 0.925, and a sensitivity of 0.714. Delong’s test for two correlated ROC curves showed no significant difference between the training and testing sets (*p* = 0.109).

**Conclusions:**

MTV_40% was a risk factor for RT-related EF in univariate analysis and was screened out using multivariate logistic regression. A model with clinical features can predict RT-related EF.

## 1 Introduction

Esophageal cancer (EC) is the fourth most common malignancy and the leading cause of cancer-related deaths in China (1, 2). Unlike the high concentration of adenocarcinoma in North America and Europe, esophageal squamous cell carcinoma (ESCC) remains the predominant malignancy in Asia (3). Due to a lack of early screening and rapid disease progression, approximately 40%–50% of patients miss the opportunity for radical surgery, which is a mainstay treatment for localized EC. Definitive chemoradiotherapy (CRT) is the standard treatment for patients with unresectable tumors or those who refuse surgery (4). For ESCC patients without distant organ metastases, except for cervical or abdominal lymph node metastases, who were treated with radiotherapy (RT) or CRT, the median overall survival was 24.3 months (5). This value was reported to be even lower (11.0 months) in a large retrospective analysis of 221 patients with advanced ESCC who developed an esophageal fistula (EF) (6).

EF is an adverse event of ESCC that develops due to tumor progression or therapeutic intervention. As one of the most serious complications of RT for EC, the incidence of EF is 1.01%–22.1% (7–15), which is 14.6%–30.5% in T4 stage patients (16–19). The clinical application of oral meglumine diatrizoate esophagogram enables early detection of EF (20), and some salvage strategies are used such as stents or bypass surgery (21, 22). However, the prognosis of EF in patients with ESCC receiving RT remains poor. The median interval from initiation of RT to the occurrence of EF was 1.3–5.75 months (7–11, 13–19), and the median post-fistula survival time is only 3.1–3.63 months (6, 7, 13). Therefore, early prediction of radiotherapy-related EF could have a significant impact on the outcome of patients with ESCC.

Fluorine-18-fluorodeoxyglucose positron emission tomography/computed tomography (^18^F-FDG PET/CT) has been widely used in patients with ESCC treated with RT in recent years. The impact of PET/CT on radiation treatment includes TNM staging of EC, optimization of RT planning, and therapeutic monitoring of neoadjuvant CRT (23). However, no study has yet investigated the ability of PET/CT scanning to detect or predict EF development, particularly for RT-related EF in ESCC patients.

In this study, we explored risk factors from clinical features and PET/CT metabolic parameters, built predictive models for RT-related EF in ESCC patients, and assessed the improvements in a model for EF prediction that combines metabolic and clinical factors over a model that incorporates only clinical features.

## 2 Methods and Materials

### 2.1 Patient Eligibility

We retrospectively collected the data of consecutive patients with ESCC treated with RT who underwent ^18^F-FDG PET/CT before treatment at our center from March 2015 to March 2021. All data were retrieved from electronic data records. The inclusion criteria were as follows: (1) squamous cell histological type, (2) treatment with RT with or without chemotherapy, (3) staging FDG PET performed before any RT or chemotherapy, (4) follow-up at least 3 months after RT or until EF was diagnosed, and (5) no EF before RT. The exclusion criteria were as follows: (1) esophageal surgery before or after RT, (2) previous thoracic RT, and (3) follow-up attrition. The institutional review board approved the retrospective analysis of routinely acquired clinical data for this study. The requirement for informed consent was waived.

### 2.2 Data Collection

Clinical data such as age, sex, Eastern Cooperative Oncology Group performance status (ECOG-PS), comorbidity, smoking/drinking history, nutritional status, TNM stage, tumor features as collected using imaging, inflammatory parameters, and EF status were collected. The metabolic parameters measured were as follows: maximum standardized uptake value (SUV_max_), mean standardized uptake value (SUV_mean_), metabolic tumor volume (MTV), total lesion glycolysis (TLG), maximum standardized uptake ratio (SUR_max_), mean standardized uptake ratio (SUR_mean_), and heterogeneity factor (HF). CT and barium meal images were reviewed by two experienced radiologists, and ^18^F-FDG PET/CT images were reviewed by two experienced nuclear medicine physicians.

#### 2.2.1 Pretreatment Evaluation

Pathological or cytological diagnosis of ESCC was confirmed by esophagoscopy. All patients were staged according to the 8th edition of the American Joint Committee on Cancer (AJCC) staging manual by endoscopic ultrasound, contrast-enhanced CT of the chest and abdomen, and ^18^F-FDG PET/CT. The T stage was diagnosed by CT when the esophagoscope could not pass through stenotic lesions in advanced disease. T3 was defined as a primary tumor with a maximum thickness of >15 mm (24). Adjacent organ invasion was defined using computed tomography (CT) or PET/CT. For example, an aortic invasion was defined as >90° of the aorta surrounded by a tumor in more than one CT slice (25), and an airway invasion was defined as deformities of the trachea or bronchi due to contiguous cancer. The maximum thickness and length of the tumor, and tumor location, were measured using PET/CT. Esophageal stenosis was quantified according to the narrowest transverse diameter identified using barium meal examination. Macroscopic tumor type was confirmed by esophagoscopy or barium meal examination according to the macroscopic classification of EC (26).

The inflammation-based parameters were platelet–lymphocyte ratio (PLR), neutrophil-to-lymphocyte ratio (NLR), monocyte-to-lymphocyte ratio (MLR), and systemic immune inflammation index (SII), which were calculated as follows: PLR = P/L; NLR = N/L; MLR = M/L; SII = P × N/L [neutrophil count (N), lymphocyte count (L), platelet count (P), and monocyte count (M)] (27). Since the records of body mass index (BMI) were lost in some patients, we had to use hemoglobin (Hb) and albumin (Alb) as indicators of nutritional status. Additionally, eosinophils, which are equipped to regulate tumor progression (28), were another candidate risk factor for EF in our study. The blood test data used in the analysis were obtained within 1 week before the initiation of treatment.

#### 2.2.2 ^18^F-FDG PET/CT Imaging and Metabolic Parameters

Pretreatment ^18^F-FDG PET/CT was performed with a time interval of <2 weeks. All patients fasted for at least 6 h (blood glucose levels below 7.0 mml/L) before PET/CT acquisition. PET/CT images were obtained 60 min later by means of a hybrid PET/CT scanner (Gemini 64 TF, Philips Medical Systems, Best, The Netherlands) after injection of 0.10–0.15 mCi/kg of ^18^F-FDG. Unenhanced CT was performed from the skull base to the mid-thigh with the following parameters: tube voltage, 120 kV; tube current, 100–110 mA; pitch, 0.829; and section and reconstruction thickness, 5 mm. After the CT scan, a three-dimensional model was used to obtain PET images, and the emission scan time for each bed position was 1–2 min. PET images were reconstructed with CT attenuation correction using the time-of-flight algorithm. Finally, all collected data were transferred into a Philips Extended Brilliance Workstation 3.0 (EBW 3.0, Philips) to reconstruct PET, CT, and PET/CT fusion images. The voxel size was 4 × 4 × 5 mm.

To calculate the SUV_max_, manually defined circular regions of interest (ROIs) were drawn on the tumor. The MTV was determined either as the total volume of voxels with a threshold SUV of 40% or as 50% of the SUV_max_ in the volume of interest. The TLG was calculated as the MTV multiplied by the SUV_mean_. The max and mean values of SUR were calculated as SUV_max_(tumor)/SUV_mean_(aorta) and SUV_mean_(tumor)/SUV_mean_(aorta) (29). To determine the HF, we first delineated the ROI with an automatic algorithm based on various SUV thresholds (e.g., 40%–80% of SUV_max_ in a 10% interval). Then, HF was calculated using linear regression analysis to identify the derivative of the volume–threshold function (30).

#### 2.2.3 Treatment

All patients in this study were treated with concurrent CRT, sequential CRT, or RT alone, 131 patients received traditional intensity-modulated radiotherapy (*IMRT*), 41 patients received volumetric modulated arc therapy (VMAT), and 13 patients underwent helical tomotherapy (TOMO).

Each patient was placed in the supine position with a head and neck thermoplastic or body vacuum bag. A planning CT (GE Healthcare UK Ltd, Amersham Place, Little Chalfont, Buckinghamshire, England) scan was performed with 0.5-cm-thick slices from the atlas (C1) to the second lumbar vertebra (L2) level. CT images were transmitted to the planning system for delineation and planning of the target area and the endangered organ. The delineation of gross tumor volume, clinical tumor volume, and planned tumor volume, and the dose and volume constraints for normal tissues, was defined according to the standard issued by the National Comprehensive Cancer Network. The IMRT and VMAT plans were developed using the Philips Pinnacle 3 software program (Philips, Amsterdam, Netherlands), and the TOMO plans were completed in the Accuray Planning Station Version 2.1.3 (TomoHD, Accuray Inc., 1310 Chesapeake Terrace Sunnyvale, CA, USA).

All eligible patients received zero to six courses of concurrent or sequential chemotherapy. The chemotherapy regimens were based on platinum, including (A) docetaxel 75 mg/m^2^ d1 or paclitaxel 135 mg/m^2^ d1 + nedaplatin 75 mg/m^2^ d2, cisplatin 75 mg/m^2^ d2, lobaplatin 50 mg d2, or carboplatin AUC 2 d2 and (B) orally S1 40 mg/m^2^ twice daily for 14 days, repeated every 3 weeks.

### 2.3 Definition of RT-Related EF

RT-related EF was defined as EF diagnosed by cervical and chest CT, barium or meglumine iothalamate esophagography of the esophagus after RT initiation, and before progression of the primary tumor. Data on all EFs after RT initiation were collected, regardless of the time interval from RT initiation. During RT, patients are routinely assessed every 3 weeks for 6 weeks by CT and X-ray esophagography. After RT, follow-up monitoring was performed once a month until the third month, then every 3–6 months until 2 years later, and annually thereafter. The types of EF (esophagorespiratory or esophagomediastinal) are described in the case report forms.

### 2.4 Statistical Analysis

The mean value comparisons of continuous variables were performed using t-tests. A chi-square test was performed to compare categorical variables. Twenty-nine clinic factors were analyzed: sex; age; ECOG-PS; smoking history; alcohol use; diabetes; macroscopic tumor type; tumor location; tumor length; maximum thickness of tumor; minimum inner diameter of tumor; TNM stage; RT fraction; RT technique; current chemotherapy; adjuvant chemotherapy (ACT); induction chemotherapy; chemotherapy regimen and circles; Hb, Alb, eosinophil, and lymphocyte counts; PLR, NLR, MLR, and SII; and 15 metabolic parameter objectives. Two multivariate prediction models were independently trained from two sets of predictors (based on clinical factors alone or based on clinical factors combined with metabolic parameters). All 185 patients were randomly divided into two cohorts in a ratio of 6:4 using computer-generated random numbers, with 111 cases in the training dataset and 74 cases in the testing dataset. Then, the training dataset was split into primary and validation sets using cross-validation-based regularization factor selection. The least absolute shrinkage and selection operator (LASSO) logistic regression was used to construct a risk score (RS) model. Receiver operating characteristic (ROC) curve analysis was conducted to evaluate the performance of predictive models and to determine the optimal RS cutoff for separating high and low risk for EF. All analyses were performed using R software (R version 4.0.2; Tableone, glmnet package, caret package, lattice package, pROC package, plyr package, ggplot2 package, foreach package, and Matrix package).

## 3 Results

### 3.1 Clinical Characteristics and Metabolism Parameters

The clinical characteristics of 185 patients are shown in **Table 1**. The median age was 63 years (range, 33–86 years). The male-to-female ratio was 4.97:1. Twenty patients (10.81%) underwent RT-related EF. Among them, eight patients experienced fistula during treatment and seven patients discontinued RT, while the other 12 developed fistula after the completion of RT. The median time of EF occurrence was 57 days (range, 5–273 days). The types of EF in this study included esophagorespiratory (three patients) and esophageal–mediastinum fistulas (17 patients). The PET/CT-based metabolism parameters are shown in **Table 2**.

**Table 1:** Clinical characteristics of 185 esophageal squamous cell carcinoma patients.

Characteristics		Esophageal fistula		*p-*value
		Without (n = 165)	With (n = 20)	
Gender (n, %)	Male	137 (83)	17 (85)	1
	Female	28 (17)	3 (15)	
Age (n, %)	<70 years	118 (71.5)	14 (70)	0.887
	≥70 years	47 (28.5)	6 (30)	
ECOG PS (n, %)	1	156 (94.5)	19 (95)	1
	2	9 (5.5)	1 (5)	
Smoking history (n, %)	No	67 (40.6)	8 (40)	0.958
	Yes	98 (59.4)	12 (60)	
Alcohol use (n, %)	No	49 (29.7)	7 (35)	0.626
	Yes	116 (70.3)	13 (65)	
Diabetes (n, %)	No	14 (8.5)	5 (25)	0.038
	Yes	151 (91.5)	15 (75)	
Macroscopic tumor type (n, %)	Protruding	60 (36.4)	5 (25)	0.373
	Ulcerative and localized	9 (5.5)	0 (0)	
	Ulcerative and infiltrative	17 (10.3)	4 (20)	
	Diffusely infiltrative	79 (47.9)	11 (55)	
Tumor location (n, %)	Cervical/upper	63 (38.2)	7 (35)	0.962
	Middle	78 (47.3)	10 (50)	
	Lower	24 (14.5)	3 (15)	
Tumor length	Median (IQR)	5 (3.6, 6.6)	6.6 (4.9, 7.7)	0.025
Tumor thickness	Median (IQR)	1.5 (1.1, 1.8)	2 (1.4, 2.3)	0.007
ID_min	Median (IQR)	0.9 (0.7, 1.2)	0.8 (0.6, 1.2)	0.882
T stage (n, %)	No–T4	89 (53.9)	6 (30)	0.043
	T4	76 (46.1)	14 (70)	
N stage (n, %)	0–1	70 (42.4)	8 (40)	0.836
	2–3	95 (57.6)	12 (60)	
M stage (n, %)	0	141 (85.5)	17 (85)	1
	1	24 (14.5)	3 (15)	
Fraction dose (n, %)	≤200 cGy	70 (42.4)	10 (50)	0.518
	>200 cGy	95 (57.6)	10 (50)	
RT technique (n, %)	IMRT	116 (70.3)	15 (75)	1
	VMAT	37 (22.4)	4 (20)	
	TOMO	12 (7.3)	1 (5)	
CCT (n, %)	No	82 (49.7)	11 (55)	0.654
	Yes	83 (50.3)	9 (45)	
Chemotherapy regimen (n, %)	No	24 (14.5)	4 (20)	0.452
	S1	10 (6.1)	2 (10)	
	TP	131 (79.4)	14 (70)	
ACT (n, %)	No	63 (38.2)	17 (85)	<0.001
	Yes	102 (61.8)	3 (15)	
ICT (n, %)	No	49 (29.7)	8 (40)	0.346
	Yes	116 (70.3)	12 (60)	
Chemotherapy circles (n, %)	0	23 (13.9)	4 (20)	0.007
	1–3	65 (39.4)	14 (70)	
	4–6	77 (46.7)	2 (10)	
Eosinophil	Median (IQR)	0.1 (0.1, 0.2)	0.1 (0.1, 0.1)	0.004
Lymphocyte	Median (IQR)	1.9 (1.5, 2.3)	1.6 (1.3, 1.8)	0.007
Hemoglobin	Median (IQR)	138 (127, 147)	136.5 (128.2, 141.5)	0.7
Albumin	Median (IQR)	40.1 (37.5, 43.2)	40.9 (37.6, 42.3)	0.915
SII	Median (IQR)	580.5 (426, 864.3)	870.6 (594, 1378.3)	0.027
PLR	Median (IQR)	135 (105.6, 169.2)	161.8 (119.1, 224.6)	0.077
NLR	Median (IQR)	2.3 (1.7, 3.1)	3.4 (2.3, 5.5)	0.005
MLR	Median (IQR)	0.2 (0.2, 0.3)	0.3 (0.2, 0.5)	0.026

IQR, interquartile range; ID_min, minimum inner diameter of tumor; IMRT, intensity-modulated radiotherapy; VMAT, volumetric modulated arc therapy; TOMO, helical tomotherapy; CCT, concurrent chemotherapy; ACT, adjuvant chemotherapy; ICT, Induction chemotherapy; SII, systemic immune inflammation index; PLR, platelet-to-lymphocyte ratio; NLR, neutrophil-to-lymphocyte ratio; MLR, monocyte-to-lymphocyte ratio.

**Table 2:** PET/CT-based metabolism parameters of 185 esophageal squamous cell carcinoma patients.

Parameters Median (IQR)	Esophageal fistula		*p*-value
	Without	With	
SUVmax	12.7 (9.1, 16.6)	13.2 (12.1, 16.2)	0.309
SUVmin_40%	4.9 (3.5, 6.2)	4.9 (4.6, 5.8)	0.446
SUVmean_40%	7.7 (5.2, 10.2)	8 (7, 9.5)	0.44
SUVsd_40%	1.9 (1.4, 2.5)	1.8 (1.6, 2.3)	0.837
MTV_40%	12.5 (7.2, 24.5)	24.9 (19.5, 34.4)	0.007
TLG_40%	100.4 (42.7, 215.7)	209.1 (106.7, 321.7)	0.01
SUVmin_50%	6.1 (4.4, 7.9)	6.5 (5.8, 7.2)	0.398
SUVmean_50%	8.5 (6.1, 11.1)	8.8 (7.7, 12.6)	0.304
SUVsd_50%	1.6 (1.2, 2.1)	1.6 (1.3, 2.2)	0.539
MTV_50%	9 (4.5, 17.6)	18.2 (13.4, 25.4)	0.007
TLG_50%	80.4 (29.3, 178.2)	164.9 (83.4, 259.7)	0.01
HF	3.7 (2, 6)	1.8 (1.5, 2.4)	0.004
SURmax	8.8 (6.1, 12.5)	9.4 (7.4, 12.4)	0.514
SURmean_40%	5.2 (3.6, 7.5)	5.5 (4.5, 7.5)	0.567
SURmean_50%	5.7 (4, 8.3)	6 (5, 8.5)	0.459

IQR, interquartile range; SUV, standardized uptake value; MTV, metabolic tumour volume; TLG, total lesion glycolysis; HF, heterogeneity factor; SUR, tumor-to-blood SUV ratio.

**Figure 1:**
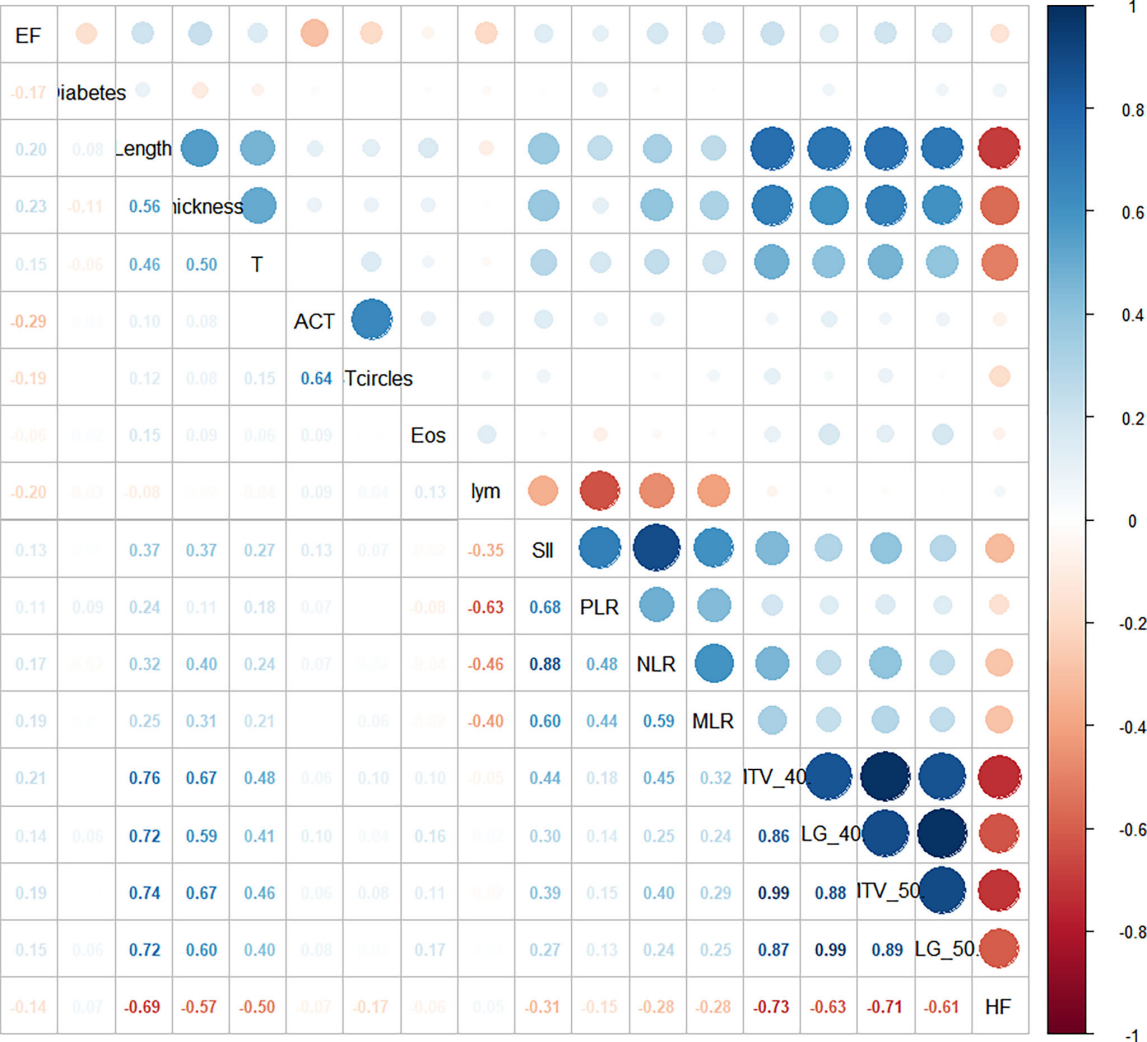
Correlation of risk factors.

Twelve of the available clinical factors and five metabolic parameters were significantly associated with RT-related EF incidence. Before logistic regression, we performed Pearson’s correlation analysis and Spearman correlation analysis of the independent variables (**Figure 1**). We found that there was high collinearity among inflammation-based parameters (absolute value of correlation coefficient, ½CC½: 0.40–0.88) and metabolism-based parameters (½CC½: 0.61–0.99). We also found a significant correlation between tumor features, such as length, thickness, T stage, and metabolism parameters (½CC½: 0.40–0.76).

### 3.2 RS Model for Radiotherapy-Related Esophageal Fistula

**Figure 2:**
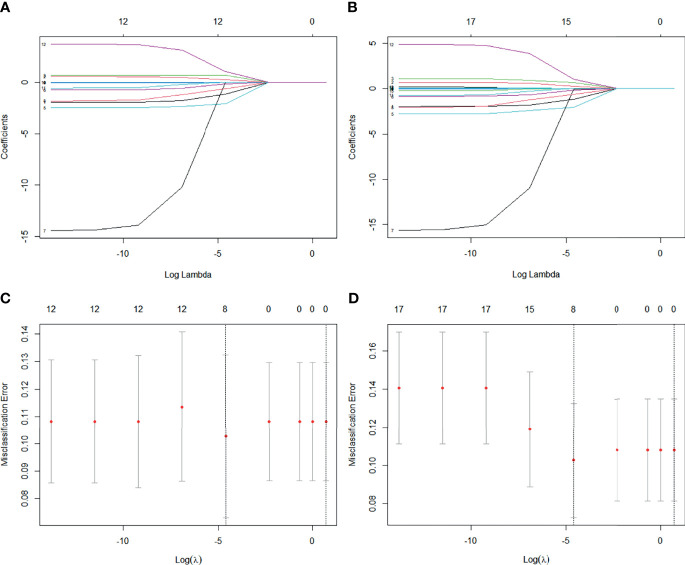
**(A)** LASSO coefficient profiles of 12 clinical features. **(B)** LASSO coefficient profiles of 12 clinical features and five metabolism parameters. **(C)** Tenfold cross-validation for tuning parameter selection in clinical features-based LASSO model. **(D)** Tenfold cross-validation for tuning parameter selection in combined features-based LASSO model.

To construct predictive models, we chose a penalized LASSO regression model to calculate an RS using the above 12 and 17 features, respectively. The LASSO coefficient profiles of 12 clinical features and 17 combined objectives in each model are shown in **Figures 2A, B**. Tenfold cross-validation was used to select an optimal model. We chose lambda.1se, a function in R, for model filtering, as shown in **Figures 2C, D**. Eight clinical features, including diabetes, tumor length, tumor thickness, adjuvant chemotherapy, chemotherapy circles, eosinophils, lymphocytes, and MLR, were selected to construct a clinical-factor-based predictive model. Only MTV_40% was added to construct a combined predictive model.

**Figure 3:**
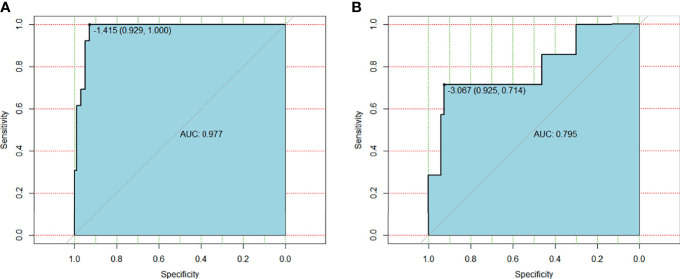
**(A)** ROC curve for clinical features based model on training group. **(B)** ROC curve for clinical features based model on testing group.

In univariate analysis, MTV_40% was a risk factor for RT-related EF, with an odds ratio (OR) of 1.036 [95% confidence interval (CI): 1.009–1.063, *p* = 0.007]. However, it was screened out from the predictive model using multivariate logistic regression analysis (**Table 3**). Finally, only one predictive model was generated using the training set based on the clinical factors. The function is as follows: RS = 0.2832 − (7.1369 × diabetes) + (1.4304 × tumor length) + (2.1409 × tumor thickness) − (8.3967 × ACT) − (28.7671 × eosinophils) + (8.2213 × MLR). The cutoff of RS in the training set was −1.415, with an area under the curve (AUC) of 0.977 (95% CI: 0.9536–1), a specificity of 0.929, and a sensitivity of 1 (**Figure 3A**). The cutoff of RS in the testing set was −3.067, with an AUC of 0.795 (95% CI: 0.577–1), a specificity of 0.925, and a sensitivity of 0.714 (**Figure 3B**). However, the Delong’s test for two correlated ROC curves showed no significant difference between the training and testing sets (*p* = 0.109).

**Table 3:** Multivariate analysis for the incidence of esophageal fistula.

Factors	Crude OR (95%CI)	Adj. OR (95%CI)	p (Wald’s test)	p (LR-test)
Diabetes	0.1 (0.02, 0.45)	0 (0, 0.14)	0.007	<0.001
Tumor length	1.32 (1.02, 1.72)	4.18 (1.64, 10.66)	0.003	<0.001
Tumor thickness	6.13 (1.82, 20.61)	8.51 (0.81, 89.63)	0.075	0.05
ACT	0.08 (0.01, 0.61)	0 (0, 0.07)	0.004	<0.001
Eosinophil	0 (0, 1.52)	0 (0, 0.02)	0.022	0.003
MLR	13.31 (0.45, 390.03)	3,719.41 (1.68, 8,232,448.12)	0.036	0.021

OR, odds ratio; CI, confidence interval; ACT, adjuvant chemotherapy; MLR, monocyte-to-lymphocyte ratio.

Assignment of involved variables were as follows: diabetes (yes = 1, no = 2), tumor length = the length of primary tumor measured on PET/CT, tumor thickness = the maximum thickness of tumor measured on PET/CT, ACT (yes = 1, no = 0), eosinophil = count number of eosinophils (×10^9^/L), and MLR = monocyte-to-lymphocyte ratio.

## 4 Discussion

EF is a severe complication in patients with ESCC treated with RT. Patients with EF experience symptoms including fever, cough, chest pain, and upper gastrointestinal bleeding. Patients may die due to sepsis or massive bleeding (31). Therefore, the prediction of radiotherapy-related EF formation is crucial before the implementation of treatment strategies. Patients who received the same dose of RT and the same intensity of chemotherapy sometimes had different outcomes of EF. This variation might be due to patient status, treatment-related factors, or tumor characteristics (12). As a functional imaging method, PET/CT can construct biological tumor volume, which could reflect cell metabolism, proliferation, hypoxia, apoptosis, and even phenotype (23). Information on tumor heterogeneity may be used to predict the occurrence of EF. Previous reports have focused on exploring the clinical risk factors of EF, while reports of metabolic parameters related to the incidence of EF are rare.

In the present study, we found that metabolic parameters, such as MTV, TLG, and HF, and diabetes, tumor length and thickness, adjuvant chemotherapy, eosinophil count, and monocyte-to-lymphocyte ratio were strongly associated with the occurrence of EF. RS models were built based on these factors.

Multiple reports have demonstrated a higher risk of radiation-induced toxicity in patients with diabetes (32, 33). However, reports on the effects of diabetes on EF are rare. Some studies tracking risk factors in EF did not show an effect of diabetes status (7, 9), while our study found that diabetes increases the risk of RT-related EF by more than seven times in ESCC.

### 4.1 Effect of Patient Status on Fistula Formation

In radiotherapy cases, a good nutritional status, such as appropriate BMI (10, 11) or serum cholesterol value (19), promotes wound healing and reduces the risk of EF formation. While the Hb and Alb levels were correlated with nutritional status in our study, there was no significant difference between the EF and non-EF groups. One possible explanation is the selection bias of the enrolled participants. Most patients who agree to perform PET/CT have the financial means to do so. Only 10 patients (5.4%) with poor performance status in our study had an ECOG-PS score of <1.

There was no significant correlation between the development of EF and tumor with ulceration or esophageal stenosis in the regression equation. This result is inconsistent with those of other studies (7–9, 12–14, 16). This finding may be due to the different assessment methods used in our study. The minimum inner diameter of the esophagus measured by barium meal examination in 117 patients was <1 cm. Since the esophagoscope could not pass through the narrowest location of the primary tumor in most cases, we had to diagnose ulceration or stenosis using radiography. We found significant differences in T4 stage, tumor length, and thickness between the groups with or without EF, which is consistent with other studies (7, 8, 11–13, 17). Finally, T4 stage was not included in the regression equation due to the correlation among these tumor characteristics.

We also focused on the effects of treatment on radiotherapy-related EF. Prior studies have reported some treatment-related risk factors, including re-RT, incomplete response, and fluorouracil-based regimens (8, 11, 12, 15). In this study, we aimed to predict the occurrence of EF before treatment delivery; therefore, factors such as treatment response after treatment were not included in the analysis. We found no significant correlation between the development of EF and RT technique and dose nor between chemotherapy regimen and circles. Unexpectedly, we found that ACT could reduce the risk of EF by more than eightfold. However, this finding does not mean that ACT could avoid RT-related EF events. A reasonable explanation may be that patients who received ACT consistently showed a poor response to RT, and the EF was most related to tumor progression.

The systemic inflammatory response has been widely used to predict the prognosis of several solid tumors, and some investors have reported that the PLR is an independent predictive indicator for EC patients who receive CRT (9). In our study, the inflammatory parameters were significantly different between with and without EF groups, and we also found a significant correlation among them. Finally, only the MLR enrolled the predictive model. Additionally, an increased eosinophil count before treatment was found to be a powerful predictor of and reduced the risk of RT-related EF. Previous reports have demonstrated that eosinophils have potent capabilities to impact local immunity and tissue remodeling during homeostasis and disease (29). The protective mechanism of eosinophils in the development of EF requires further research.

To the best of our knowledge, this study is the first to investigate the relationship between PET/CT-based metabolism parameters and the development of RT-related EF. The MTV, TLG, and HF were different between the with and without EF groups, and there was a significant correlation among them. Only MTV_40% was selected as a risk factor to construct a predictive model after LASSO analysis and was finally screened out by multivariate logistic regression. A reasonable explanation is that MTV_40%, which is the total volume of voxels with a threshold SUV of 40% of the SUV_max_ in the volume of interest, is highly correlated with the size of the tumor. This will reduce the ability of MTV to present the heterogeneity of the tumor. Radiomics is a recent area of research in precision medicine and is based on the extraction of a large variety of features from medical images. PET radiomics may be a promising approach for predicting the development of EF instead of metabolic parameters.

The present study had several limitations. First, this was a retrospective study conducted at a single institution. Second, the sample size was small, and inherent biases were inevitable. Third, external validation of the predictive model should be performed in the future.

## 5 Conclusion

We failed to construct a predictive model for RT-related EF in ESCC patients combined with PET/CT-based metabolism parameters. However, we developed an RS model integrating patient characteristics, tumor and treatment-related factors, and inflammatory parameters. This model might help to discriminate high-risk populations in clinical practice that are susceptible to RT-related EF and individualize treatment plans to prevent it.

## Data Availability Statement

The raw data supporting the conclusions of this article will be made available by the authors, without undue reservation.

## Ethics Statement

The studies involving human participants were reviewed and approved by the Ethics Committee of Fujian Medical University Cancer Hospital. Written informed consent for participation was not required for this study in accordance with the national legislation and the institutional requirements.

## Author Contributions

KL and XN designed the study and wrote the manuscript, recruited patients, collected data, and did statistical analysis. These two authors contributed equally. JL provided idea of this research. DL extracted the PET/CT-based metabolism parameters. All authors contributed to the article and approved the submitted version.

## Funding

This study was funded by a grant from the Quanzhou City Science & Technology Program of China (2018N049S).

## Conflict of Interest

The authors declare that the research was conducted in the absence of any commercial or financial relationships that could be construed as a potential conflict of interest.

## Publisher’s Note

All claims expressed in this article are solely those of the authors and do not necessarily represent those of their affiliated organizations, or those of the publisher, the editors and the reviewers. Any product that may be evaluated in this article, or claim that may be made by its manufacturer, is not guaranteed or endorsed by the publisher.
