# Supplementary figures and images for: Incorporation of PET Metabolic Parameters With Clinical Features Into a Predictive Model for Radiotherapy-Related Esophageal Fistula in Esophageal Squamous Cell Carcinoma
Source: Front Oncol. 2022 Feb 28;12:812707. doi: 10.3389/fonc.2022.812707 (PMC8918510; doi:10.3389/fonc.2022.812707)

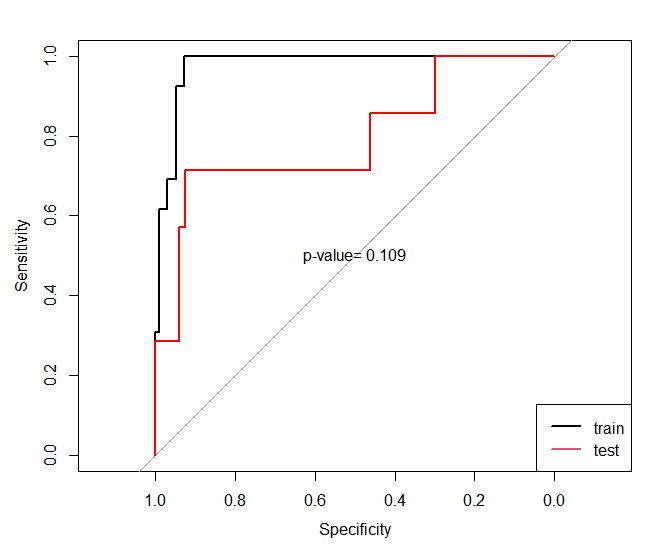

Supplement: Supplementary file 3 [file Image_1.tiff]
